# Optical Imaging Demonstrates Tissue-Specific Metabolic Perturbations in Mblac1 Knockout Mice

**DOI:** 10.1109/JTEHM.2024.3355962

**Published:** 2024-01-15

**Authors:** Busenur Ceyhan, Parisa Nategh, Mehrnoosh Neghabi, Jacob A. LaMar, Shalaka Konjalwar, Peter Rodriguez, Maureen K. Hahn, Matthew Gross, Gregory Grumbar, Kenneth J. Salleng, Randy D. Blakely, Mahsa Ranji

**Affiliations:** Biophotonics LaboratoryDepartment of Electrical Engineering and Computer Science, College of Engineering and Computer ScienceFlorida Atlantic University 1782 Boca Raton FL 33431 USA; Department of Biomedical ScienceCharles E. Schmidt College of MedicineFlorida Atlantic University 1782 Boca Raton FL 33431 USA; Stiles-Nicholson Brain Institute, Florida Atlantic University 1782 Jupiter FL 33458 USA; Division of Research, Comparative MedicineFlorida Atlantic University 1782 Boca Raton FL 33431 USA

**Keywords:** MBLAC1, Alzheimer’s disease, optical metabolic imaging, redox state, metabolic syndrome

## Abstract

Objective: Metabolic changes have been extensively documented in neurodegenerative brain disorders, including Parkinson’s disease and Alzheimer’s disease (AD). Mutations in the C. elegans swip-10 gene result in dopamine (DA) dependent motor dysfunction accompanied by DA neuron degeneration. Recently, the putative human ortholog of swip-10 (MBLAC1) was implicated as a risk factor in AD, a disorder that, like PD, has been associated with mitochondrial dysfunction. Interestingly, the AD risk associated with MBLAC1 arises in subjects with cardiovascular morbidity, suggesting a broader functional insult arising from reduced MBLAC1 protein expression and one possibly linked to metabolic alterations. Methods: Our current studies, utilizing Mblac1 knockout (KO) mice, seek to determine whether mitochondrial respiration is affected in the peripheral tissues of these mice. We quantified the levels of mitochondrial coenzymes, NADH, FAD, and their redox ratio (NADH/FAD, RR) in livers and kidneys of wild-type (WT) mice and their homozygous KO littermates of males and females, using 3D optical cryo-imaging. Results: Compared to WT, the RR of livers from KO mice was significantly reduced, without an apparent sex effect, driven predominantly by significantly lower NADH levels. In contrast, no genotype and sex differences were observed in kidney samples. Serum analyses of WT and KO mice revealed significantly elevated glucose levels in young and aged KO adults and diminished cholesterol levels in the aged KOs, consistent with liver dysfunction. Discussion/Conclusion: As seen with C. elegans swip-10 mutants, loss of MBLAC1 protein results in metabolic changes that are not restricted to neural cells and are consistent with the presence of peripheral comorbidities accompanying neurodegenerative disease in cases where MBLAC1 expression changes impact risk.

## I. Introduction

In 2015, 46.8 million individuals worldwide had dementia, with projections doubling to 74.7 million by 2030 [1]. In 2020, the World Health Organization reported 55 million dementia cases worldwide, with Alzheimer’s disease (AD) accounting for 70% [2]. Alzheimer’s disease, the primary cause of dementia, is becoming one of the most expensive, deadly, and burdensome diseases of the 21st century [3]. The causes of AD are not fully understood, but it appears to involve a combination of genetic, environmental, and lifestyle factors.

The complexity of AD is revealed by various theories [4]. The development of AD is associated with cholinergic deficiency [5], amyloid beta ( $\text{A}\beta$) toxicity [6], tau protein hyperphosphorylation [7], synaptic dysfunction [8], oxidative stress [9], and neuroinflammation [10]. All these factors accelerate the symptoms of AD’s progression regardless of the root cause. Sporadic Alzheimer’s disease is the most prominent type that develops after the age of 65 and is commonly linked to metabolic illnesses including Type 2 diabetes, brain trauma, and lifestyle habits [11], [12]. Familial Alzheimer’s disease typically appears earlier in life and is caused by hereditary genetic mutations in amyloid precursor protein (APP), presenilin 1 (PSEN1), and presenilin 2 (PSEN2) [13], [14], [15]. These mutations are dominant and highly penetrant and lead to an accumulation in the brain of $\beta $-amyloid protein [14], [15]. Extensive research acknowledges the significant role of non-genetic factors in initiating AD, but only a limited number of these factors have been identified.

The nematode *Caenorhabditis elegans* has proven to be a powerful model for identifying molecules essential for neuronal development, signaling, health, and degenerative diseases due to its conserved molecular architecture with vertebrates [16], [17], [18], [19]. The nematode is particularly useful as a platform for unbiased forward genetic screens where mutants that impact cellular and organismal function can be isolated in a few days. Such efforts require the availability of a readily quantifiable phenotype following worm mutagenesis or suppression of RNA expression (e.g. RNAi). Mutations in the *C. elegans* presynaptic dopamine (DA) transporter (*dat-1*) have been shown to produce a phenotype termed Swimming-Induced Paralysis (Swip) when transferred from a solid substrate to water [20], [21]. Using Swip as a screen for genes that control DA signaling, Hardaway et al. identified a novel gene, labeled *swip-10*. These investigations showed that the DA-dependence of paralysis in *swip-10* mutants arises from excess DA secretion that extrasynaptically inhibits the worm motor program [22]. Follow-up studies revealed the Swip to arise in these animals from glutamate (Glu) overstimulation of DA neurons, with evidence consistent with changes in glial-dependent Glu clearance that leads to excess Glu activation of excitatory Glu receptors [23]. GFP reporter fusions revealed the *swip-10* gene to be expressed in glial cells, consistent with alterations in glial support for synaptic Glu homeostasis. Continued investigations with *swip-10* mutants demonstrated premature degeneration to be specifically associated with glial unsheathed neurons, including DA neurons [24], consistent with glial contributions to human neurodegenerative diseases. As with Swip, DA neuron degeneration in *swip-10* mutants involves glial-dependent, excess stimulation of Glu receptors [24]. However, these receptors were found to be distinct from the Glu receptors that cause Swip, being ones that flux Ca^2+^, and suggesting a contribution of altered Ca^2+^ homeostasis to *swip-10-* induced neurodegeneration [24]. More broadly, *swip-10* mutants demonstrated systemic oxidative stress, as indicated by increased expression of a reactive oxidative species (ROS) sensitive reporter (*gst-4*: GFP) [24].

Phylogenetic sequence alignments identified a metallo- $\beta $-lactamase domain (MBD) containing protein MBLAC1 as the putative mammalian ortholog of SWIP-10 [25]. Notably, replicated genome-wide association studies support altered *MBLAC1* expression or function as a risk factor for Alzheimer’s disease with peripheral cardiovascular comorbidity (AD-CVD) [26]. A follow-up post-mortem study demonstrated a reduction of *MBLAC1* mRNA in the frontal cortex of AD-CVD subjects [26]. Peripheral comorbidity for disorders linked to *MBLAC1* is consistent with evidence of peripheral expression of *MBLAC1* and prior *Mblac1* KO mouse serum studies, which revealed alterations in multiple metabolic pathways [25] and an oxidative stress phenotype [23].

Intrinsic fluorophores such as reduced nicotinamide adenine dinucleotide (NADH), and oxidized flavin adenine dinucleotide (FAD) can be used to evaluate the intracellular metabolic redox state and mitochondrial function [27]. In the mitochondrial electron transport chain (ETC), electron donors FADH_2_ and NADH support the production of ATP, and reactive oxygen species (ROS) are produced as a natural byproduct. Oxidative stress (OS) occurs due to the inability of cells to detoxify excess amounts of ROS or the loss of the cellular antioxidant defense [28]. Increased production of ROS and OS results in a more oxidized redox state, as published previously [29], [30]. NADH and FAD quantitation can be used to deduce metabolic activity since metabolic potential coincides with the ratio of reduced vs oxidized substrates [30]. Importantly, *MBLAC1* has been linked to AD, which shows evidence of altered mitochondrial function and oxidative stress [31]. Evaluating metabolic activity in peripheral tissues may explain why reduced *MBLAC1* is a risk factor for AD with peripheral comorbidity [26].

Optical imaging is a highly effective and precise diagnostic tool that differentiates diseased and healthy tissue. Its simple methodology and ability to generate quality data make it well-suited for various applications. Optical metabolic imaging measures the auto-fluorescence of NADH and FAD. The approach is used to generate a redox ratio (RR), NADH/FAD, a quantitative biomarker used to determine metabolic differences between healthy and disease models [32], [33], [34]. The ratiometric measurement of these metabolites in tissue cancels out system noise and makes the RR independent of the number of mitochondria in the tissue. 3D optical cryo-imaging utilizes low temperatures (−10°) to elevate the quantum yield of the fluorophores NADH and FAD. A high signal acquisition is crucial in constructing 3D images showing global metabolic activity [35]. Additionally, the harvesting practice of rapid freezing with isopentane and liquid nitrogen preserves the tissue’s metabolic state by trapping the steady state of electron transfer in cytochromes [35]. Chance et al. verified that the fluorescent signal of NADH and FAD originates from the mitochondria with negligible contributions from the cytosol [36]. Upon a metabolic perturbation, the auto-fluorescence signal from NADPH is significantly lower and produces minor changes compared to the NADH auto-fluorescent signal [37], [38], [39]. Similarly, the FADH_2_ cofactor is non-auto fluorescent [40], [41]. Accordingly, the NADH and FAD intensities are direct reflections of mitochondrial activity. Optical imaging sheds light on metabolic activity that can be translated to inform and develop mitochondria-targeted therapeutics for neurodegenerative diseases.

Our study used optical imaging to investigate metabolic/mi tochondrial deficits in the peripheral tissues of *Mblac1* KO mice. Biomarkers NADH, FAD, and RR were quantified with optical cryo-imaging to assess the metabolic state of isolated livers and kidneys from adult WT and KO mice of both males and females. Through these efforts, we establish altered, sex-independent, and tissue-specific metabolic deficits arising as changes in altered levels of key cofactors involved in mitochondrial ATP synthesis. Serum analysis provides evidence of glucose elevations in young and old adult KO mice and lower cholesterol levels in older animals, consistent with liver dysfunction and metabolic abnormalities like those in Type 2 diabetes.

## II. Materials and Methods

### A. Animal Models

Homozygous *Mblac1* KO mice [25] and their wild type (WT) littermates were produced from heterozygous breeders maintained at Florida Atlantic University (FAU), Jupiter campus. Animals were housed, bred, and studied under an approved protocol reviewed by the FAU Institutional Animal Care and Use Committee (IACUC). The *Mblac1* KO mice studied were of mixed sex, were 10 weeks old, and were bred from heterozygous mutant parents. The KO mice possess a homozygous deletion of 5 base pairs in exon 1 of the *Mblac1* gene, leading to a frameshift in early coding sequences of the protein, thereby eliminating protein expression. Genotyping of mice was performed on tail snip-derived DNA (Transnetyx, Inc., Cordova, TN). The total number of samples for WT and littermate KO is 16 (N=8/group, 4 male, 4 female) for the livers and 36 (N=18/group, 11 male, 7 female) for the kidneys. The tissues were submerged in liquid isopentane before being quickly frozen in liquid nitrogen to reduce structural distortions and cracks caused by direct exposure to liquid nitrogen. Frozen tissues were stored at −80°C before analysis. For serum collection, 9-14 weeks old (N=9-10/group) and 18-20 months old (N=6/group) mice were euthanized via CO2 asphyxiation in compliance with the AVMA Guidelines for Euthanasia of Animals. Immediately after euthanasia, mice and tissues were weighed, and blood was collected via cardiocentesis. Blood was placed in BD Microtainer serum separator tubes (Becton Dickinson, Franklin Lakes, NJ). The samples were centrifuged, and the serum was submitted for clinical pathology to an outside laboratory (VRL Diagnostics, Gaithersburg, MD) for sampling of blood urea nitrogen (BUN), cholesterol, glucose, insulin, alkaline phosphatase (ALP), creatine phosphokinase (CPK) and creatinine.

### B. 3D Optical CRYO-Imaging

**FIGURE 1.**
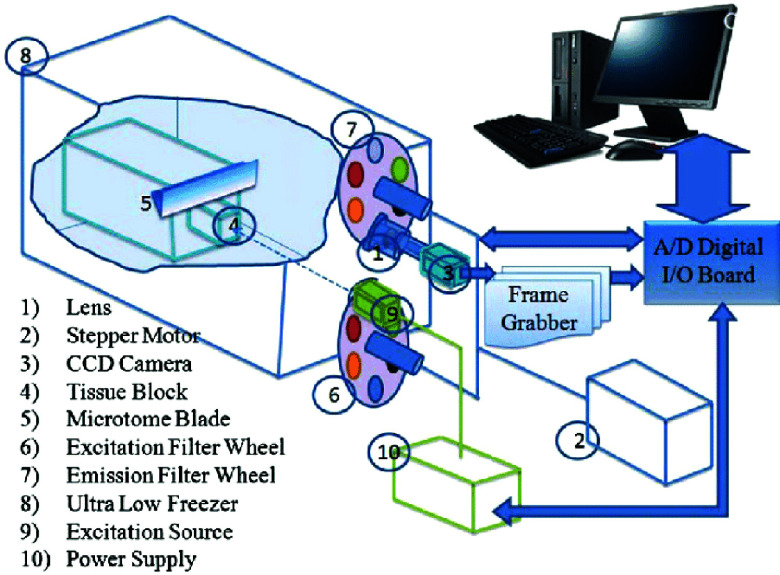
A schematic is shown of the automated image acquisition cryo-microtome, the cryo-imager. Numbers 1-12 depict each component of the automated system. Surface of tissue is imaged in two channels (NADH and FAD) at each successive slice.

The Biophotonics Lab (FAU, Boca Raton, FL) designed a 3D fluorescence cryo-imager (Figure 1). This instrument is designed to take photos of fluorophores and slice tissue sequentially. Blinded to sex and genotype, tissues were embedded one day before imaging in a black absorbent optical cutting temperature medium. To image, the frozen tissue block was mounted to the sample stage, where its temperature was maintained at −10° during image acquisition. Cryogenic temperatures of −10° allowed for a higher quantum yield of fluorescence signals while retaining markers of tissue metabolic state. A motor-driven stage and microtome blade allowed tissue slicing at $100\mu \text{m}$ (liver) and $30\mu \text{m}$ (kidney) thickness. The cryo-imager has alternating filter wheels to capture NADH and FAD and acquires images using a CCD camera (Retiga R6, Teledyne Photometrics, Tucson, AZ). Tissue fluorophores were excited with a mercury arc lamp (200W lamp, Oriel, Irvine, CA). The broad light passes through excitation filters 350 ± 40 nm (UV Pass Blacklite, HD Dichroic, Los Angeles, CA) for the NADH channel and 437 ± 10 nm (440QV21, Omega Optical, Brattleboro, VT) for the FAD channel. The emission filters on the imaging end of the instrument are set at 460 ± 25 nm (D460/50M, Chroma, Bellows Falls, VT) for the NADH channel and 537 ± 50 nm (QMAX EM 510-560, Omega Optical, Brattleboro, VT) for the FAD channel. Filter wheels, microtome blade, and image acquisition operate with an automated virtual interface LabVIEW software (National Instruments, Austin, TX). Previous reports describe the instrument and experiment protocol in detail [27], [33], [34].

### C. Imaging Processing

MATLAB R2022b (MathWorks Inc., Natick, MA) was used to process NADH and FAD autofluorescence images. 200 images from NADH and FAD channels were captured for tissues (livers and kidneys). Calibrations and pre-processing were performed to account for discrepancies from day-to-day imaging. Variables such as light intensity, illumination pattern, and dark current noise were considered when processing. A segmentation algorithm based on thresholding was applied to each slice to segment the region of interest of the tissue. The images were stacked in the z-direction to generate 3D images. The RR was calculated by dividing the fluorescence values of NADH over FAD images voxel by voxel.

The redox ratio distribution over the whole tissue is presented in histograms. The mean values of these histograms were calculated using Equations (1) and (2). The histograms provide information on the intensity distribution within the respective tissues.

\begin{align*} &\hspace {-.1pc}Mean \\ &=\frac {1}{N_{x}\times N_{y}\times N_{z}}\!\sum \nolimits _{i=1}^{N_{x}} \!\sum \nolimits _{j=1}^{N_{y}}\! \sum \nolimits _{k=1}^{N_{z}} {Liver\_{}Volume(i,j,k)} \tag{1}\\ &\hspace {-.1pc}Mean \\ &=\frac {1}{N_{x}\times N_{y}\times N_{z}}\!\sum \nolimits _{i=1}^{N_{x}} \!\!\sum \nolimits _{j=1}^{N_{y}} \!\sum \nolimits _{k=1}^{N_{z}}\! {Kidney\_{}Volume(i,j,k)} \tag{2}\end{align*}

### D. Statistical Evaluation of 3D Optical CRYO-Imaging Data

Data are reported as mean ± standard error (SEM). Statistical comparisons were conducted on the mean values of image histograms using a two-way analysis of variance (ANOVA). ANOVA was performed to evaluate variance, the significance of differences in fluorescence components, and their relative contributions among the WT and KO groups. The significance level for genotype, sex, and their interactions were determined with p < 0.05. When sex-independent results were evident via a two-way ANOVA, males, and females were combined to enhance the statistical power for analysis via Student’s t-test.

## III. Results

**FIGURE 2.**
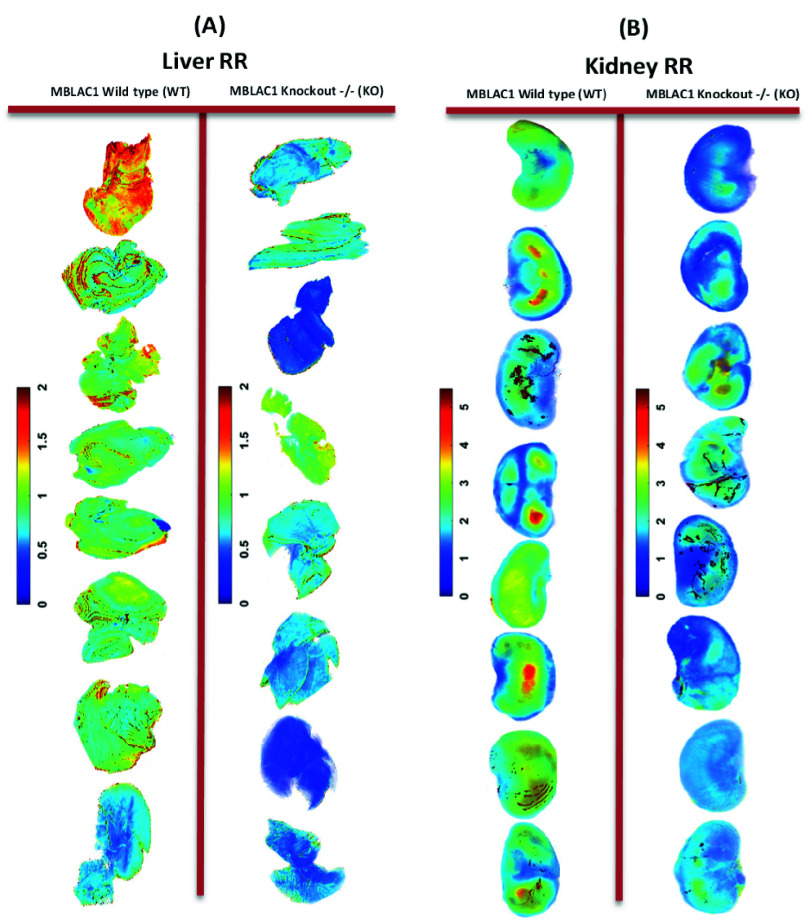
3D reconstructions of WT and KO groups. Panel (A) shows RR (NADH/FAD) for all 16 livers (male and female) and panel (B) shows 16 representative kidneys (male and female). Scale bars coincide with RR and its distribution is revealed in the respective tissues. Red indicates a higher RR value thus a more reduced state. In contrast, blue indicates a lower RR and a more oxidized state.

Normalized NADH, FAD, and RR intensities were calculated to evaluate the metabolism and effectiveness of the *Mblac1* mutation. Figure 2 presents the RR of WT and KO groups across 3D volumes of tissue, 16 whole livers (A), and 16 representative whole kidneys (B). The intensity of the NADH/FAD signal is reflected by the pseudo-colored scaling bar, where blue reflects a low intensity and red reflects a high intensity. Tissue closer to the red reflects a more reduced state, while blue reflects a more oxidized state.

**FIGURE 3.**
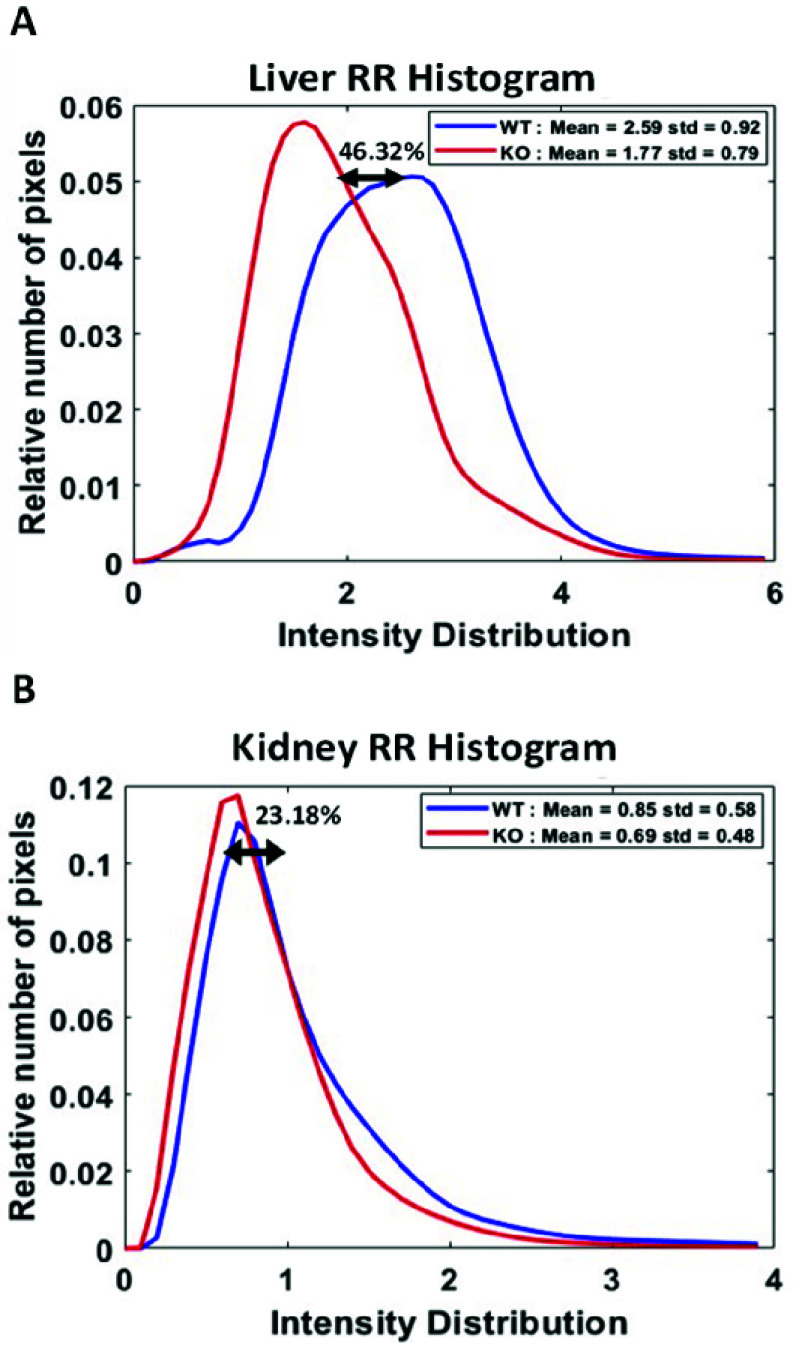
Histograms represent mean RR for all KO and WT livers (A) and kidneys (B). Red designates KO, while blue designates WT values. The mean values of WT and KO livers are 2.59 ± 0.92 and 1.77 ± 0.79. Kidney values are 0.85 ± 0.56 for WT and 0.69 ± 0.48 for KO. Both KO livers and kidneys present a decrease in mean value, with a 46.32% reduction observed for livers and a 23.18% reduction observed for kidneys.

Histogram plots present RR for all livers (Figure 3A) and all kidneys (Figure 3B) of WT vs KO groups. The tissue histograms are a scaled density function of RR intensities voxel by voxel, and their calculations are described in the Methods (*C. Image Processing*). The mean values depict the degree of reduced dinucleotide coenzymes involved in mitochondrial ATP production. A lower mean value indicates a less reduced and more oxidized state, whereas a higher mean value indicates a more reduced and less oxidized state. RR for WT and KO of livers revealed mean values of 2.59 ± 0.92 and 1.77 ± 0.79, respectively, resulting in a 46.32% decrease in RR for KO livers compared to WT. Kidney RR mean values were 0.85 ± 0.56 for WT and 0.69 ± 0.48 for KO yielding a reduced yet non-significant change (23.18%).

**FIGURE 4.**
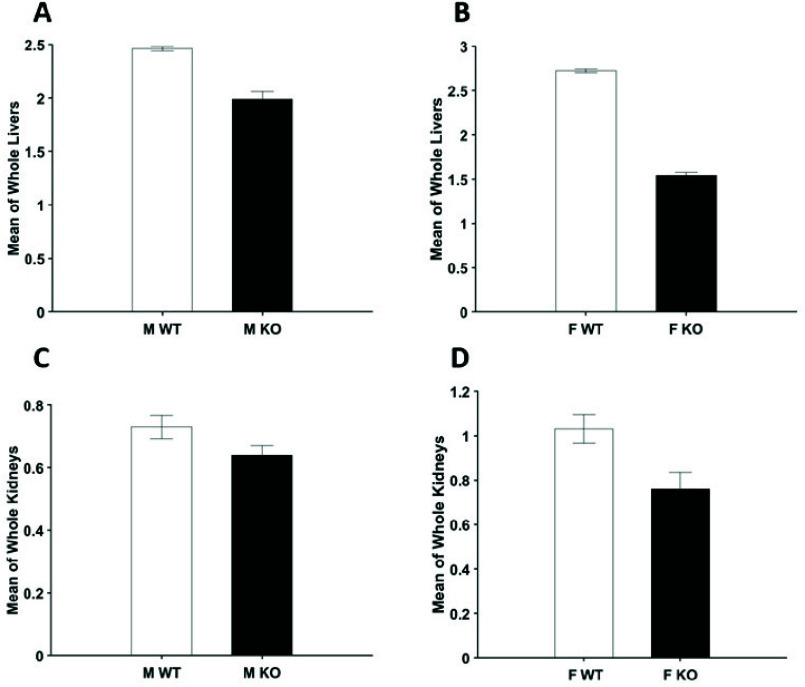
Bar graphs present mean value of the RR ± SEM histograms for male (A) and female (B) livers and male (C) and female (D) kidneys. Two-way ANOVA was carried out for a total of 16 WT and KO livers (4 male, 4 female per group) and 36 WT and KO kidneys (11 male, 7 female per group). Significant sex-dependent differences and genotype x sex interactions were not observed for livers and kidneys.

A two-way ANOVA was performed to test for sex and genotype as variables for RR on tissues of WT and KO. As we did not observe a sex effect or sex x genotype interaction, we combined sexes to increase power and performed a two-sample t-test. As shown in Figure 4A, liver samples demonstrated a significant reduction in RR in KO groups. Kidney samples did not show significance, though a mean reduction in RR was also observed.

**FIGURE 5.**
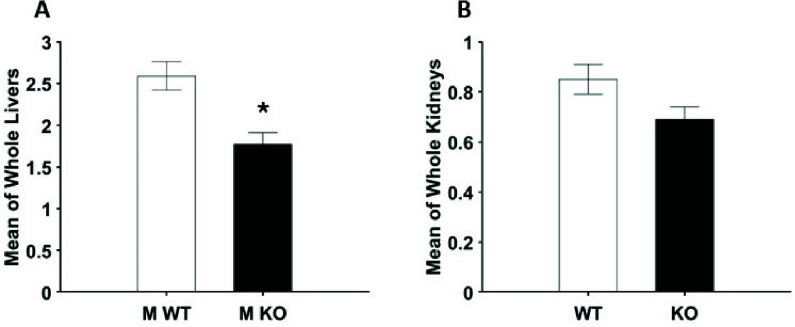
Bar graphs show the mean value of the RR ± SEM histograms for livers (A) and kidneys (B). Student’s t-test of mean values of RR was carried out for livers (N = 16) and kidneys (N = 36). (A) A significant difference for livers (one-sided Student’s t-test, p = $0.021 < 0.05$) was found between groups. The significant difference is marked with $^{\ast} $. (B) Significant difference for kidneys (p = 0.17 > 0.05) was not found between the groups.

Mean RR values were calculated for WT and KO’s livers and kidneys (male and female combined). Figure 5 presents a statistical analysis using a One-sided Student’s t-test for liver (A) and kidney (B) groups. We observe a significant variation between WT and KO groups for livers. In contrast, the same test on kidneys reported insignificant variation.

**FIGURE 6.**
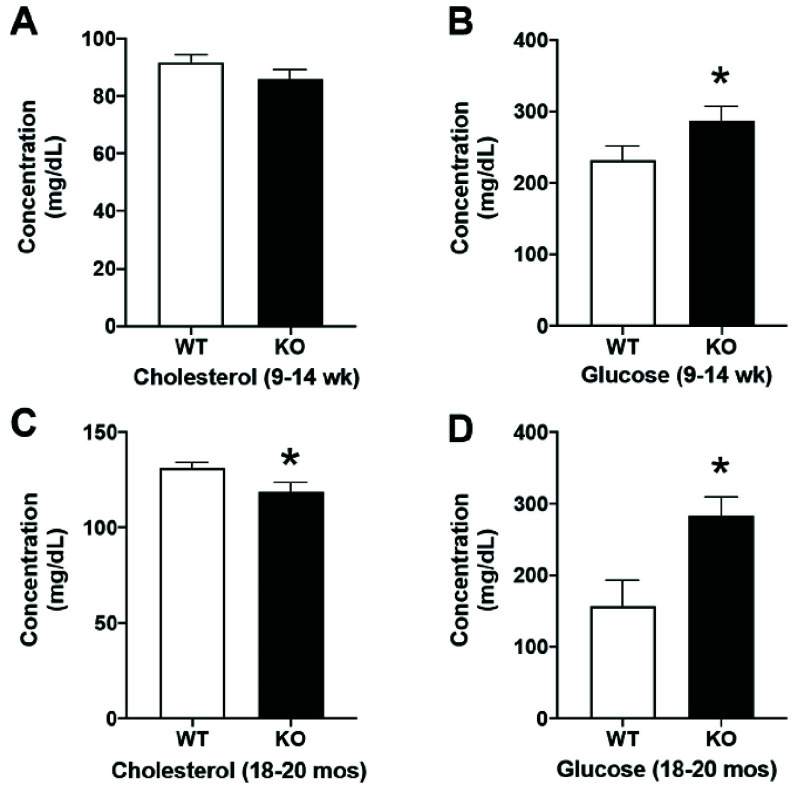
Bar graphs compare genotypic effects in 9-14 weeks old mice for cholesterol (A) and glucose (B), and in 18-20 months old mice for cholesterol (C) and glucose (B) via Student’s t-test (p < 0.05). Significant differences are reported in figures B, C, and D with a $^{\ast} $.

Given the RR findings noted above, we performed further complementary analyses on tissues and serum from 9-14 weeks old WT and KO mice. No genotype differences were found in body weight nor the weight of most organs, including liver and kidney. Conspicuous was a significant, sex-independent, 11.5% elevation in heart weight (Student’s t-test, p < 0.05). In serum, no genotype changes were evident in creatinine, CPK, ALP, or insulin levels. We did detect a significant, sex-independent elevation of BUN levels (17.5%, Student’s t-test, p < 0.01). Given the prominent role played by the liver in cholesterol and glucose homeostasis, we evaluated these measures as well. Whereas no genotype effects were seen for cholesterol (Figure 6A), glucose levels in the KO displayed a significant, sex-independent elevation (23.8%, Figure 6B). In a small group of older animals (18-20 mos), we repeated the latter two measures, finding now a small but significant, sex-independent decrease in cholesterol levels (Figure 6C) and a much more significant increase (79.9%) in glucose levels (Figure 6D).

## IV. Discussion

RR, NADH/FAD, obtained from optical metabolic imaging, was employed to evaluate tissue mitochondrial redox state, an important determinant of mitochondrial bioenergetics, in a model of AD risk, *Mblac1* KO mice versus their WT littermates. Our evidence reveals a decrease in RR for KO liver and kidney groups, as observed from the 3D volumetric RR (Figure 2). The mean value of the RR in the liver and kidney decreased by 46.32% and 23.18% in KO, respectively. The histograms (Figure 3) align with 3D volumetric data, highlighting a contrast between the two groups. The oxidized status of *Mblac1* KO expresses a redox-mediated oxidative modification at the cellular level. Our research shows that the *Mblac1* KO model possesses substantial vulnerability to maintaining redox homeostasis. Our RR measurements point to ROS-mediated oxidative stress [42], [43], which is well-known to be involved in the onset of neuronal disorders like AD [31]. Determining *Mblac1*’s role in mitochondrial dysfunction is vital for understanding how mitochondrial bioenergetics pairs with neurodegeneration and developing mitochondrial-targeted therapeutics. Recent studies from the Blakely lab in the *C. elegans* model system have revealed that loss of the gene *swip-10*, the closest worm homolog to *Mblac1*, regulates the production of cuprous copper, Cu(I), with deletion of the gene triggering a loss of mitochondrial function, ATP production and oxidative stress (Rodriguez et al., *submitted*) [44]. Studies are underway to assess Cu(I) homeostasis in *Mblac1* KO mice.

ROS is widely recognized to be responsible for oxidative stress-mediated cumulative damage to cellular components [45] and is noted as necessary for AD propagation [46], [47], [48], [49]. The site of ROS production is in the mitochondria and is marked by electron leakage during electron transfer [50]. Oxidative phosphorylation (OxPhos) occurs in the mitochondrial electron transport chain (ETC) and majorly contributes to redox homeostasis and mitochondrial ROS metabolic enzymes. Perturbations to critical players of OxPhos have previously been linked to AD pathology [46], [51]. Electron leakage occurs via two mitochondrial carriers of the ETC, NADH for complex I and FAD for complex II, two dominant sites of ROS generation [52]. The two coenzymes provide the ETC free energy and electrons, build the proton gradient, and drive ATP synthesis. When mitochondria are overly active, they are oxidized and are demonstrated by low NADH levels and high FAD levels [53]. Notably, NADH/FAD ratios are commonly employed to imply fluctuations in ROS levels [42], [43]. Panels for KO (Figure 2) exhibit a global skew of NADH and FAD, inferring a decrease in the pairing of electrons with hydrogen and increased leakage of electrons to available oxygen. When cells lose this steady state, unstable and highly reactive ROS threatens mitochondrial integrity [54]. Glycolysis and the tricarboxylic acid cycle (TCA) produce NADH and FAD, contributing to the pool of these coenzymes. In addition, lipid peroxidation, protein oxidation, and DNA/RNA oxidation are all caused by ROS attacks and progress AD pathology [31].

There is strong evidence for a link between the development of AD, diminished mitochondrial function, and oxidative stress [46], [47], [48], [49]. The oxidized redox state exhibited in *Mblac1* KO mice supports this narrative. Oxidative stress is revealed by cumulative damage to proteins, lipids, and other cellular components caused by excess ROS generation [45], [50]. It is noted that AD is linked to the exceeding insult to nucleic acid bases [55]. The damage caused to DNA surpasses cellular repair and detoxification methods. DNA-repairing enzymes like glycosylases and apurinic/apyrimidinic (AP) endonucleases experience a depleting expression rate, making it exceedingly difficult to counter oxidatively damaged nucleic acids [56]. Additionally, lipid peroxidation, recognized to be a marker of excess ROS, gives rise to numerous reactive neurotoxic agents, among which are 4-hydroxy-trans-2-nonenal (HNE) [57], [58] and 4-oxo-trans-2-nonenal (4-ONE) [58]. When these compounds react with amino groups of proteins in the carbonyl group, they form Schiff bases and Michael additions [59]. These reactions inevitably cause protein inactivation because of the protein crosslinks. Michael reactions at the sulfhydryl group of cysteines can progenerate DNA damage due to cross-linking [59]. HNE, a lipid peroxidation product, is heavily accredited to progressing AD [58], [59]. HNE and 4-ONE maintain the structure integrity of protein disulfide isomerase through the reaction at sulfhydryl groups [59]. In general cases, glutathione (GSH), a ROS buffer with antioxidant properties, can attenuate damage inflicted by HNE and 4-ONE [60], [61]. Consistent with this model, our past [24] and ongoing studies with *swip-10* worm mutants revealed elevations in oxidative stress reporters, including altered GSH homeostasis.

The 3D optical cryo-imaging tool was used in this study to observe the redox state by fluorescent signals NADH, and FAD is a powerful method to evaluate mitochondrial changes. We have shown that the loss of expression of MBLAC1 protein in mice leads to an oxidized redox state in the liver. These findings support a critical role for MBLAC1/SWIP-10 in producing reduced dinucleotide coenzymes essential for mitochondrial ATP production and explain the contribution of diminished MBLAC1 expression to AD risk if observed in the CNS. Notably, the mice used in our studies were young adults, indicating that changes arise earlier than expected for an AD risk factor. This suggests that reduced MBLAC1 expression combined with human etiological factors may pose an increased risk for AD progression in early adulthood or later in life. Metabolic deficits present in young adulthood contribute to the development of AD and its comorbidities when other risk factors become penetrant. Prior metabolomics studies on serum from *Mblac1* KO mice also revealed a change in oxidative stress-linked metabolites (e.g., elevated ascorbic acid) and reduced bile acid levels produced in the liver. Bile acids are a product of cholesterol biosynthesis, and here, we show that serum cholesterol levels are reduced in older *Mblac1* KO mice. We also found a significant elevation in BUN levels. An elevation in BUN has been reported in non-alcoholic fatty liver disease (NAFLD), which is associated with AD [62] and nominated as a biomarker for cardiovascular disease [63]. Notably, changes in serum glucose were evident in young and old adult mice without changes in insulin levels, reminding us of the correlation between type II diabetes and metabolic syndrome in Alzheimer’s disease [64]. The form of AD linked to *MBLAC1* expression has cardiovascular disease comorbidity [26]. Although the basis for this association is at present unknown, the elevated heart weight we observe in the *Mblac1* KO model may reflect a compensatory response of tissue with high ATP demand; altogether, these findings support a tissue-specific metabolic role for MBLAC1 protein and support *Mblac1* as a genetic risk factor for AD [26] with peripheral comorbidity.

Our research focuses on expanding our knowledge of the mechanisms responsible for AD progression so that we can guide mitochondria-mediated interventions to minimize or delay the risk of AD. Future studies will examine severe and non-severe genetic mutations and potential treatment options. Based on previous research [22], [23], [24], [25], pharmacological treatments that restore normal metabolic function via manipulation of *Mblac1* pathways may be of benefit in the treatment of neurodegenerative disorders, including AD and Parkinson’s disease. The cryo-imaging protocol used here can be extended to interrogate these disorders to evaluate better metabolic changes leading to disease risk and the development of new therapies.

## V. Conclusion

Using optical cryo-imaging, our results present quantitative differences of mitochondrial redox state in WT and Mblac1 KO tissues (liver and kidney). Specifically, we report significant differences in the RR of WT and KO liver. A reduced RR status for KO groups suggests that loss of *Mblac1* interferes with the biochemical machinery dictating OxPhos, which is expected to decrease ATP production and increase ROS-mediated oxidative stress. Mitochondrial dysfunction has been reported in AD, and thus, our data presents a case where the oxidized redox state established by loss of MBLAC1 protein expression could sensitize neurons to AD pathological mechanisms, particularly those targeting mitochondria. Our findings of altered glucose and cholesterol homeostasis, along with changes in heart mass, suggest the presence of a metabolic syndrome that may drive the cardiovascular dysfunction associated with AD who demonstrate reduced *MBLAC1* expression.
